# A comparison of the effectiveness of cervical medial branch radiofrequency ablation for chronic facet joint syndrome in patients selected by two common medial branch block paradigms

**DOI:** 10.1016/j.inpm.2022.100091

**Published:** 2022-04-08

**Authors:** Taylor R. Burnham, Nathan Clements, Aaron Conger, Keith Kuo, Joshua Lider, Marc Caragea, Richard Kendall, Shellie Cunningham, James B. Meiling, Masaru Teramoto, Zachary L. McCormick

**Affiliations:** a Department of Physical Medicine and Rehabilitation, University of Utah, Salt Lake City, UT, USA; b Department of Physical Medicine and Rehabilitation, University of Texas Health Science Center San Antonio, San Antonio, TX, USA; c School of Medicine, University of Utah, Salt Lake City, UT, USA; d Department of Physical Medicine and Rehabilitation, Mayo Clinic, Rochester, MN, USA

**Keywords:** Facet-mediated pain, Cervical spine, Radiofrequency ablation, Medial branch blocks, Protocol guidelines

## Abstract

**Background:**

Cervical medial branch radiofrequency ablation (CMBRFA) is effective when patients are selected by dual medial branch blocks (MBBs). SIS guidelines recommend 100% pain improvement after dual comparative MBBs before CMBRFA; however, our prior investigation showed similar outcomes in those selected by a lesser strict paradigm.

**Objective:**

Compare pain and patient impression of improvement after CMBRFA in individuals stratified by a less stringent (80–99%) dual MBB response than those selected by the 100% criteria.

**Design:**

Cross-sectional study.

**Methods:**

Follow-up was conducted via standardized telephone survey at ≥6 months post-CMBRFA to obtain Numerical Rating Scale (NRS) pain and Patient Global Impression of Change (PGIC) scores. Primary and secondary outcomes were within-group and between-group differences in the proportions of patients reporting ≥50% NRS score reduction and PGIC scores.

**Results:**

Medical records of 195 consecutive patients were reviewed; 100 individuals were analyzed. 48% (95% CI 35–61%) and 52% (95% CI 37–67%) of the 80–99% and 100% MBB groups, reported ≥50% pain reduction at ≥6 months post-CMBRFA. 74% (95% CI 63–85%) and 67% (95% CI 52–81%) of the 80–99% and 100% MBB groups reported a PGIC score consistent with “improved” or “very much improved.” There were no significant between-group differences in any outcome at any time point.

**Conclusions:**

We observed similar rates of pain relief and global improvement after CMBRFA in patients selected by dual MBBs with ≥80% symptom relief versus 100% relief. This provides evidence that a more practical criteria, compared to a more strict selection paradigm, may result in similar clinical outcomes.

## Abbreviations

cervical medial branch radiofrequency ablation: (CMBRFA)
medial branch blocks: (MBB)
numerical rating scale: (NRS)
patient global impression of change: (PGIC)
lumbar medial branch radiofrequency ablation: (LMBRFA)
radiofrequency ablation: (RFA)
minimally clinical important change: (MCID)
medial branch nerve: (MBN)
confidence interval: (CI)

## 1 Introduction

Neck pain is among the most commonly reported musculoskeletal complaint in the United States, with a significant worldwide burden [1]. The cervical zygapophysial joints, more colloquially known as “facet joints,” have a 25–45% reported pain prevalence [2,3]. Cervical facet pain is diagnosed by medial branch blocks (MBBs). Such blocks also provide predictive value for cervical medial branch radiofrequency ablation (CMBRFA), improving both pain and function [2,4]. The efficacy of CMBRFA was established in the late 1990's [5]. Early studies utilized the strict patient selection criteria of 100% symptom resolution with multiple diagnostic MBBs. As such, the Spine Intervention Society (SIS) recommends that patient selection be limited to individuals who experience 100% pain relief after at least two concordant MBBs with the rationale to reduce false-positive rates [[5], [6], [7], [8], [9], [10]].

Since the 1990's, many providers have adopted less stringent patient selection criteria based on supporting literature for diagnosing lumbar facet joint pain. Today, many insurances require ≥80% pain improvement after two diagnostic MBBs before qualifying for lumbar medial branch radiofrequency ablation (LMBRFA); thus, the less stringent lumbar MBB criteria have been adopted by providers as the diagnostic and CMBRFA selection criteria in the cervical spine. There is minimal literature comparing outcomes stratified by the historically strict and now common selection paradigms. Our preliminary work demonstrated no significant outcome differences after CMBRFA in participants selected with the strict (100%) versus the more relaxed (80–99%) dual block criteria, suggesting the strict patient selection criteria may prevent patients from receiving an effective treatment pain-relieving intervention [11].

The purpose of the present study was to expand upon our preliminary investigation comparing the effectiveness of CMBRFA utilizing patient selection criteria of ≥80% symptom improvement with dual MBBs in a larger patient cohort.

## 2 Methods

### 2.1 Data collection

This cross-sectional cohort study was conducted at a tertiary academic spine center. Local Institutional Review Board (IRB 00107596) approval was obtained. The electronic medical records of consecutive patients who underwent CMBRFA between August 8, 2012, to March 2, 2020, were reviewed. Inclusion criteria were: (1) age ≥18 years old; (2) cervical neck pain ≥3 months; (3) documented ≥80% symptom improvement with dual MBBs at anatomic levels determined by clinical suspicion before CMBRFA; (4) ≥6 months between CMBRFA and survey; and (5) willingness to participate in a post-procedural phone call survey. Data extraction was performed by authors (T.B., A.C., S.C., K.K., J.L, and M.C.). Data collected included the following: (1) age; (2) sex; (3) body mass index; (4) presence of cervical fusion; (5) highest recorded numeric rating scale (NRS) neck pain score within two months before CMBRFA; (6) percentage of pain improvement with each MBB; (7) date of CMBRFA; (8) CMBRFA level(s) and laterality; (9) CMBRFA probe type (cooled or conventional radiofrequency ablation [RFA] electrode); and (10) number of previous CMBRFAs. Patients who met the inclusion criteria were contacted via a letter sent by their treating physician regarding the research project. Patients completed a post-CMBRFA phone call survey which captured a last-7-day average NRS pain score and self-reported improvement by the Patient Global Impression of Change (PGIC).

### 2.2 Outcomes

The primary outcome was the proportion of patients with ≥50% pain reduction at least six months after the most recent CMBRFA. Secondary outcomes included (1) the proportion of patients who reported being “much improved” or “very much improved” (PGIC scores 6–7) and (2) the proportion of patients who reported ≥ 2-point NRS reduction from baseline, which is the minimal clinically important change (MCID) for neck pain [12]. The between-group comparisons of each outcome were calculated for those selected for CMBRFA by 80–99% relief versus 100% relief after dual MBBs.

### 2.3 Procedures

All MBBs and CMBRFA procedures were performed by physicians fellowship-trained in interventional spine procedures who are faculty members at the University of Utah.

### 2.4 Medial branch blocks

Patients were positioned in the lateral recumbent position, ipsilateral to the laterality of the MBB. The skin was prepped and draped in a sterile fashion. The skin was anesthetized with approximately 1 ​mL of 1% lidocaine at each site. Using a lateral fluoroscopic approach, 25-gauge, 1.5–2.5 inch short bevel needles were advanced to the appropriate anatomic landmarks, identifying the TON, C3–C7 medial branch nerves (MBNs). Correct needle placement was confirmed with an anteroposterior (AP) view on fluoroscopy. Next, approximately 0.1–0.3 ​mL of contrast medium was injected at each site to ensure proper needle placement and rule out intravascular injection. After correct needle placement was confirmed, 0.3–0.5 ​mL of either 4% lidocaine or 0.5% bupivacaine was injected. Throughout the dual MBBs, each patient received lidocaine and bupivacaine (either anesthetic during each MBB), but the order depended on provider preference. All patients were blinded to the local anesthetic. Post-procedurally, patients were provided a pain log and instructed to document symptom improvement in 15-min increments for 6 hours using the NRS. Positive response to MBBs was defined as ≥80% pain reduction on two separate occasions.

### 2.5 Radiofrequency ablation - conventional and cooled

For patient safety, intravenous access and cardiopulmonary monitoring were established. For conventional CMBRFA, the patient was positioned prone on the procedure table, the skin was prepped, and the patient was draped in the usual sterile fashion. The skin and underlying soft tissues were anesthetized with 2–3 ​mL of 1% lidocaine at each site. Then an 18-gauge introducer needle was advanced under AP fluoroscopic imaging to the C2-3 joint line for the third occipital nerve, to the centroid of the lateral mass for the C3-6 MBNs, and the superior/anterior portion of the lateral mass for the C7 MBN. Precise needle placement was confirmed with AP, oblique, and lateral fluoroscopic imaging. The needles were advanced to the anterior margin of the lateral pillar but posterior to the adjacent neuroforamen. Care was taken to ensure the active tip of the cannula was positioned parallel to the expected course of the MBN, as described in SIS practice guidelines [7]. After confirming appropriate electrode positioning, approximately 2 ​mL of 2% lidocaine was injected for anesthesia during the ablation. Then, an RFA probe with a 10-mm active tip (Baylis Medical, Montreal, Canada) was inserted into the introducer. Two RFA lesions were performed over each of the MBNs. For the third occipital nerve, a lesion was created slightly superior and slightly inferior to the C2–C3 joint. For the C3–C6 MBNs, a lesion was created once along an oblique path to target the nerve as it crossed the lateral aspect of the pillar and again at a 30-degree sagittal approach to target the nerve over the anterior lateral aspect of the pillar. For the C7 MBNs, one lesion was created at the superior aspect and another lesion at the inferior aspect of the triangular superior articular process of C7. Each lesion was heated to 80 ​°C for 90 seconds.

For cooled CMBRFA, the patient was positioned in a lateral recumbent position and the skin was prepped, and the patient was draped in the usual sterile fashion. Next, a 17-gauge introducer needle was advanced under a lateral fluoroscopic view to contact the appropriate anatomic landmarks for the third occipital nerve, C3-6 MBNs and C7 MBN, respectively, as described above. An 18-gauge probe with a 2–4 ​mm active tip (Coolief Cooled Radiofrequency Kit, Halyard Health, Alpharetta, Georgia) was inserted perpendicular to the MBN, given the forward projection of cooled RFA lesions beyond the active tip of the electrode [13]. After correct needle placement was confirmed in AP and lateral fluoroscopic views, each MBN was anesthetized with approximately 2 ​mL of 2% lidocaine. Radiofrequency lesioning was then carried out for 150 seconds at a generator setting of 60 ​°C (>80 ​°C intralesional temperature) after a 30-s ramp-up time.

### 2.6 Statistical analysis

Descriptive statistics were calculated for patient demographic, clinical, and procedure-related variables. Whether or not patients experienced ≥50% pain relief, and improvement of ≥2 points on the NRS (MCID), and a score of ≥6 on the PGIC was summarized using frequencies and percentages. A 95% confidence interval (CI) was calculated for each percentage, and an exact binomial test was used for examining statistical significance. These outcome variables were also stratified by MBB response (80–99% vs. 100%), separately for 6–12, 12–24, and >24 months post-CMBRFA, and were analyzed using a two-by-two contingency table analysis. The relative risk (RR) of treatment success (defined above) with a 95% CI and Fisher's exact *p*-value were calculated for each contingency table analysis. Further, numeric changes in pain before and after CMBRFA were examined using a dependent *t*-test, separately by MBB response (80–99% vs. 100%). Binary logistic regression analysis with the calculations of RR and 95% CIs were performed to explore the relationship between the primary outcome of ≥50% pain reduction at minimum six-month follow-up and a covariate of interest, namely the type of CMBRFA (conventional vs. cooled).

## 3 Results

Of the 195 consecutive patients identified by a database query, 95 were excluded due to repeat CMBRFA procedure since the telephone survey began August 8, 2012 (n ​= ​26), lack of complete MBB documentation (n ​= ​47), and refusal of participation or inability to contact the patient (n ​= ​22). Thus, a total of 100 patients were included in the final analysis.

Patient demographic, clinical, and procedure-related variables are described in Table 1*.* Fifty-eight percent and 42% of patients reported 80–99% and 100% symptom improvement, respectively, after dual MBBs before CMBRFA. Sixty-two percent, 29%, and 9% of outcomes were captured at 6–12, 12–24, and >24 months post-CMBRFA, respectively. Seventy-nine and 21% of patients received cooled or conventional CMBRFA, respectively. Seventy-eight percent of patients reported this as their first treatment with CMBRFA.

**Table 1:** Patient demographics and procedure-related variables.

Quantitative variable		Mean (SD)
Age (yr)		57.6 (14.4)
Body mass index (kg/m^2^)		29.1 (7.0)
**Categorical variable**		**Frequency (%)**
Gender	Male	46 (46.0)
	Female	54 (54.0)
Follow-up time	6–12 months	62 (62.0)
	12–24 months	29 (29.0)
	≥24 months	9 (9.0)
Dual MBB response	80–99%	58 (58.0)
	100%	42 (42.0)
Fusion	No fusion	93 (93.0)
	Fusion	7 (7.0)
Duration of pain	<1 year	17 (17.0)
	1–5 years	48 (48.0)
	≥5 years	35 (35.0)
Side	Left	38 (38.0)
	Right	28 (28.0)
	Both	34 (34.0)
TON	Yes	23 (23.0)
	No	77 (77.0)
Number of levels denervated	1	46 (46.0)
	2	43 (43.0)
	3	11 (11.0)
Trainee present	None	86 (86.0)
	Resident	2 (2.0)
	Fellow	11 (11.0)
	Resident and fellow	1 (1.0)
Repeat CMBRFA	Yes	22 (22.0)
	No	78 (78.0)
CMBRFA type	Traditional	21 (21.0)
	Cooled	79 (79.0)

MBB ​= ​Medial branch block, TON ​= ​Third occipital nerve, CMBRFA = Cervical medial branch radiofrequency ablation.

Cumulative post-CMBRFA outcomes reported for NRS pain scores and PGIC scores are summarized in Table 2. Forty-eight percent (95% CI 35–61%) and 52% (95% CI 37–67%) of the 80–99% and 100% MBB groups, respectively, reported ≥50% pain reduction at a minimum of 6 months post-CMBRFA. Sixty-seven percent (95% CI 55–79%) and 64% (95% CI 50–79%) of the 80–99% and 100% MBB groups, respectively, reported ≥2 point NRS pain reduction (NRS MCID) pain reduction at a minimum of 6 months post-CMBRFA. Seventy-four percent (95% CI 63–85%) and 67% (95% CI 52–81%) of the 80–99% and 100% MBB groups, respectively, reported a PGIC score consistent with 6 (“improved”) or 7 (“very much improved”) at follow-up.

**Table 2:** Pain and patient impression of change outcomes stratified by medial branch block response.

Outcome variable	Time since RFA	Dual MBB response	Yes	No	RR (95% CI)	*p*
≥50% NRS pain score reduction	6–12 months	80–99%	16 (43.2)	21 (56.8)	0.83 (0.49–1.41)	0.498
		100%	13 (52.0)	12 (48.0)		
	12–24 months	80–99%	9 (56.3)	7 (43.7)	1.04 (0.54–2.03)	0.897
		100%	7 (53.9)	6 (46.1)		
	≥24 months	80–99%	3 (60.0)	2 (40.0)	1.20 (0.36–4.04)	0.999§
		100%	2 (50.0)	2 (50.0)		
	Minimum of 6 months	80–99%	28 (48.3)	30 (51.7)	0.92 (0.62–1.36)	0.685
		100%	22 (52.4)	20 (47.6)		
≥2 point NRS pain score reduction	6–12 months	80–99%	23 (62.2)	14 (37.8)	0.97 (0.66–1.43)	0.883
		100%	16 (64.0)	9 (36.0)		
	12–24 months	80–99%	12 (75.0)	4 (25.0)	1.22 (0.73–2.04)	0.688§
		100%	8 (61.5)	5 (38.5)		
	≥24 months	80–99%	4 (80.0)	1 (20.0)	1.07 (0.52–2.18)	0.999§
		100%	3 (75.0)	1 (25.0)		
	Minimum of 6 months	80–99%	39 (67.2)	19 (32.8)	1.05 (0.78–1.40)	0.758
		100%	27 (64.3)	15 (35.7)		
≥6 on PGIC	6–12 months	80–99%	26 (70.3)	11 (29.7)	0.98 (0.71–1.35)	0.883
		100%	18 (72.0)	7 (28.0)		
	12–24 months	80–99%	13 (81.3)	3 (18.7)	1.32 (0.81–2.16)	0.406§
		100%	8 (61.5)	5 (38.5)		
	≥24 months	80–99%	4 (80.0)	1 (20.0)	1.60 (0.55–4.68)	0.524§
		100%	2 (50.0)	2 (50.0)		
	Minimum of 6 months	80–99%	43 (74.1)	15 (25.9)	1.11 (0.86–1.45)	0.416
		100%	28 (66.7)	14 (33.3)		

RFA ​= ​Radiofrequency Ablation, MBB ​= ​Medial Branch Block, PGIC = Patient Global Impression of Change, RR ​= ​Relative risk, CI = Confident interval, NRS = Numeric Rating Scale.

§ From Fisher's exact test.

**Fig. 1:**
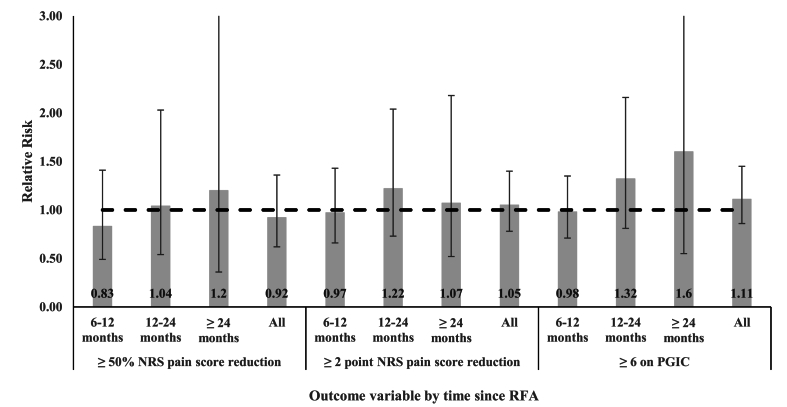
Relative risks (80–99% vs. 100% pain relief after dual MBBs) for outcomes at different time points.

There were no significant between-group differences observed in the two MBB groups (80–99% vs. 100% relief) for any outcome measure (≥50% pain reduction, ≥2 points NRS pain reduction, PGIC of 6 or 7) at any time point (6–12, 12–24, or >24 months) (Fig. 1). All of the 95% CI of the RR values overlapped with 1.00, indicating that the rates of relief after CMBRFA were not significantly different the patients selected by 100% relief after dual MBBs versus those selected with the 80% relief threshold. The binary logistic regression model showed that there was no significant difference in conventional vs. cooled CMBRFA in predicting ≥50% pain reduction (RR ​= ​0.89, 95% CI [0.34–2.32], p ​= ​0.81).

No serious adverse effects or complications related to the dual MBBs or CMBRFA were identified via database review or phone call follow-up.

## 4 Discussion

In this cross-sectional study of consecutive patients with ≥80% relief upon dual MBBs who were subsequently treated with CMBRFA, approximately 50% experienced ≥50% reduction of pain, approximately 70% achieved or exceeded the NRS MCID for neck pain, and approximately 70% reported a PGIC score of at least “much improved” at a minimum of six months post-CMBRFA. There were no significant between-group differences in the MBB groups (80–99% vs. 100% relief) for any outcome measure at any time point. The 95% CI for the RR of treatment success across all outcomes overlapped 1.00, indicating that the probability of success was not significantly different between the MBB groups. These findings suggest that in routine clinical practice, outcomes after CMBRFA may not differ substantially when patients are selected with 80–99% versus 100% relief threshold.

The results of this study confirm the findings of our earlier work [11]. Before our two studies, no prior research has compared post-CMBRFA outcomes in patients selected with ≥80% improvement after dual MBBs to those chosen with the strict selection criteria of 100% pain reduction at dual MBBs within a single study as used in the early research and as found in the current SIS guidelines. A 2020 systematic review by Engel et al. [14] concluded that the post-CMBRFA responder rate would be highest in patients with 100% pain reduction after dual MBBs; however, this conclusion was derived by comparing study responder rates, not within study group comparison. Without proper statistical analysis, between-study outcome comparisons are a increased risk of confounding variables (i.e., different patient demographics, CMBRFA techniques, etc.). The current studies’ results are reassuring to clinicians and patients, knowing that the current and more relaxed patient selection for CMBRFA (≥80% improvement after dual MBBs) is no different from the more rigid selection criteria (100% after dual MBBs). The potential impact of these results includes increased access to CMBRFA for patients that original selection criteria would have excluded.

Though there was no between-group difference in outcomes based on MBB selection criteria, the proportion of responders that achieved ≥50% pain reduction after CMBRFA was lower than those found in the original seminal research studies [5,8]. A 2016 systematic review of CMBRFA by Engel et al. reported that 63% of patients were pain-free at six months and 38% were pain-free at one year when patients were selected with the recommended SIS selection criteria of 100% symptom improvement with dual MBB, with or without placebo block (149/238, 95% CI [57–69%], vs. 86/226, 95% CI [32–44%]) [16]. However, only 10% (10/100) of the participants in the current study experienced 100% pain reduction in the present study. Interestingly, seven of the ten participants with 100% pain relief were in the group of patients that had 80–99% symptom improvement with dual MBBs, meaning that none of these patients would have been selected for any of the studies included in Engel et al.'s systematic review in 2016 [15].

There may be multiple factors other than MBB response responsible for the reduced responder rates in each MBB group. Some notable possibilities include technical changes and the limitation of the current study. Since the seminal research [5,8,9,16], multiple techniques and technologies have been developed; however, no significant, well-designed, comparative study has assessed their effectiveness. The patients included in the original studies were treated with conventional RFA technology, which involved a parallel approach to the RFA procedure and required multiple nerve lesions. Conversely, the patients in the current study were treated primarily with water-cooled RFA (79%), which generally involves a perpendicular approach with a single, large forward-projecting lesion. Though the regression analysis showed no between-group outcome differences, the study was not powered to identify these differences. Although RCTs have shown similar effectiveness between cooled RFA versus conventional RFA in lumbar spine [17,18], to our knowledge, no study has compared these technologies in the cervical spine.

The authors recognize that the utilized CMBRFA patient selection criteria (≥80% improvement after dual MBBs) may be stricter than those recommended or used in other societies or practices. The providers in the current study used the referenced selection paradigm because it best aligned with the SIS guidelines and local insurance requirements (requiring ≥80% pain improvement after dual MBBs). Recently, an international workgroup published a consensus practice guideline on interventions for cervical joint pain [19]. The group recommended that CMBRFA be done in patients who experience ≥50% pain reduction after a single MBB. It should be noted that these recommendations are based on a minimal number of studies (three studies [two retrospective and one underpowered observational study], excluding the work done by this group) [[20], [21], [22]]. The overall responder rates in the current study are similar to those in the consensus guideline referenced work. Despite similar responder rates, readers should exercise caution when comparing outcomes across studies with different patient demographics or interventional techniques. Our opinion is that additional higher-quality, large, prospective research is needed to better understand the ideal selection criteria.

The study design is a limitation of the current study. Generally, the causal relationship of an independent variable/exposure/intervention (e.g., MBB block response) and a dependent variable/outcome (e.g., post-CMBRFA pain reduction) is established when variables are tested in a specific population free of bias, confounders, or chance. The most effective way to establish a causal relationship or interventional efficacy is done in a large, blinded, randomized controlled trial or a large, well-controlled cohort study. Such study designs significantly reduce bias, confounding effects, and make the reader more confident in the relationship between an intervention and outcome [[23], [24], [25], [26]]. Less rigorously designed studies reduce the causal relationship's confidence and the interaction between the independent and dependent variables-the relationship is referred to as an association and not causal. The current study is a cross-sectional study because the independent and dependent variables were evaluated simultaneously with varying, between patient, outcome time points. The cross-sectional data in the present study helps determine the prevalence of participants who obtained the reported outcome at a specific point in time but does not reveal the cumulative incidence of subjects who experienced the primary and secondary outcomes at some other time point. Such limitations introduce potential information bias and potential confounding effects. Any co-interventions between the intervention and outcome survey may have been responsible for the patient's reported response. These were not monitored or restricted.

## 5 Future research

Given the limitations of this cross-sectional study, future research with prospective collection of clinical outcome data controlled for confounders is essential in determining the effectiveness of CMBRFA when stratified by various block paradigms. Studies comparing the efficacy of cooled perpendicular, single-lesion RFA and conventional parallel, multi-lesion RFA in the cervical spine would be beneficial.

## 6 Conclusions

In this cross-sectional study, the largest to date in a population from the United States (n ​= ​100), we observed similar rates of pain relief and global improvement after CMBRFA in patients selected by dual MBBs with ≥80% symptom relief versus 100% relief. This study suggests that a more practical, rather than a more strict, selection paradigm may result in similar clinical outcomes.

## Funding source

This work was supported by The Skaggs Clinical Spine Research Seed Grant Program at the 10.13039/100007747University of Utah.

## Declaration of competing interest

The authors declare the following financial interests/personal relationships which may be considered as potential competing interests: Zachary L. McCormick, MD reports a relationship with Spine Intervention Society that includes: board membership. Zachary L. McCormick, MD reports a relationship with Avanos Medical US that includes: funding grants.
